# Orange Pomace and Peel Extraction Processes towards Sustainable Utilization: A Short Review

**DOI:** 10.3390/molecules28083550

**Published:** 2023-04-18

**Authors:** Qingxiao Li, Nicky Rahmana Putra, Dwila Nur Rizkiyah, Ahmad Hazim Abdul Aziz, Irianto Irianto, Lailatul Qomariyah

**Affiliations:** 1 College of Grain Engineering, Henan Industry and Trade Vocational College, Zhengzhou 451191, China; 2 Centre of Lipid Engineering and Applied Research (CLEAR), Ibnu Sina Institute for Scientific and Industrial Research, Universiti Teknologi Malaysia, Johor Bahru 81310, Malaysia; 3 Faculty of Food Science and Nutrition, Universiti Malaysia Sabah, Kota Kinabalu 88400, Malaysia; 4 Department General Education, Faculty of Resilience, Rabdan Academy, Abu Dhabi 114646, United Arab Emirates; 5 Department of Industrial Chemical Engineering, Institut Teknologi Sepuluh Nopember, Surabaya 60111, Indonesia

**Keywords:** orange, peels, pomace, limonene, pectin, phenolic, green extraction

## Abstract

More than 58 million metric tonnes of oranges were produced in 2021, and the peels, which account for around one-fifth of the fruit weight, are often discarded as waste in the orange juice industry. Orange pomace and peels as wastes are used as a sustainable raw material to make valuable products for nutraceuticals. The orange peels and pomace contain pectin, phenolics, and limonene, which have been linked to various health benefits. Various green extraction methods, including supercritical carbon dioxide (ScCO_2_) extraction, subcritical water extraction (SWE), ultrasound-assisted extraction (UAE), and microwave-assisted extraction (MAE), are applied to valorize the orange peels and pomace. Therefore, this short review will give insight into the valorization of orange peels/pomace extraction using different extraction methods for health and wellness. This review extracts information from articles written in English and published from 2004 to 2022. The review also discusses orange production, bioactive compounds in orange peels/pomaces, green extractions, and potential uses in the food industry. Based on this review, the valorization of orange peels and pomaces can be carried out using green extraction methods with high quantities and qualities of extracts. Therefore, the extract can be used for health and wellness products.

## 1. Introduction

Waste management has evolved from emissions reduction or mitigation towards a more comprehensive strategy. For sustainable growth, waste materials must be seen as valuable commodities that can be used as raw resources. Limiting natural resource use to attain a balanced standard is associated with a reduction in greenhouse gas emissions, which have a negative impact on climate change. Agro-industrial wastes are related to gas pollution from processing, usage, and disposal. They contribute to the depletion of natural resources as a result of a growing global population [1,2]. The new concepts of “circular economy” and “cradle-to-cradle” attempt to develop trash from certain industrial processes that may be utilized as sustainable raw materials. These principles would highlight vital environmental and economic challenges regarding the utilization of renewable resources [3,4,5].

Fruit juice industries produce about 5% lignin, 9% cellulose, and 80% hemicellulose and create enormous amounts of trash. Since they are biodegradable, they create leachates and methane with biochemical and chemical oxygen demands, resulting in increased recovery costs. Since 2017, our group has been valorizing agricultural wastes to move towards a “zero-waste” economy to obtain high value products, reduce emissions, and contribute to the healthier production of components and commodities. One of the research projects related to the valorization of agricultural waste regards the extraction of peanut skins. In order to recover catechin, antioxidant, and flavonoid components, peanut skins have been extracted using ScCO_2_ extraction as a sustainable technology. This technology successfully recovers high concentrations of the bioactive compounds, especially for catechin and epicatechin (752.03 µg/g and 3399.84 µg/g, respectively) with CO_2_ and food-grade ethanol, a non-toxic solvent [6]. Additionally, banana peels are extracted using a sustainable process known as SWE [7]. This technology successfully extracts a high yield of low-methoxyl pectin (LMP) and high-methoxyl pectin (HMP) with shorter extraction times compared to conventional extraction [7].

**Figure 1:**
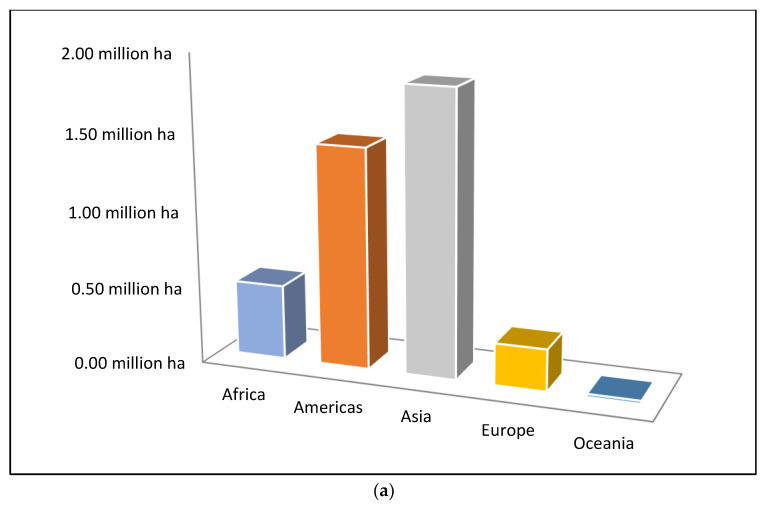

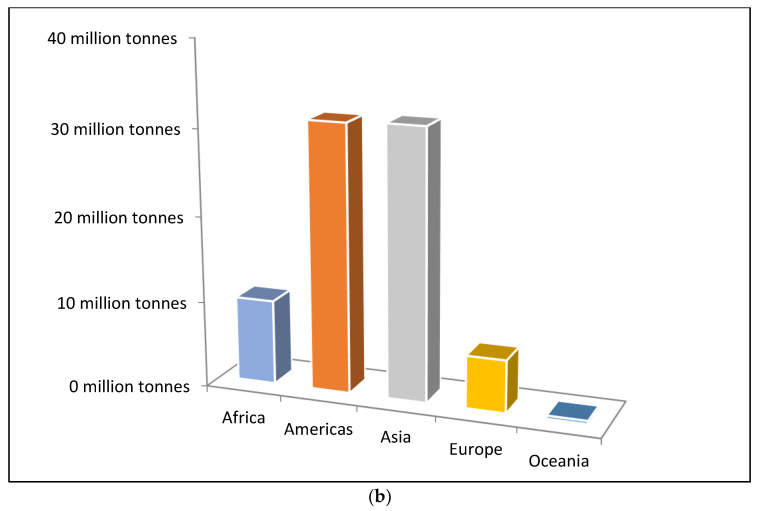
Harvested area (**a**) and production (**b**) of oranges in 2021 [8].

**Figure 2:**
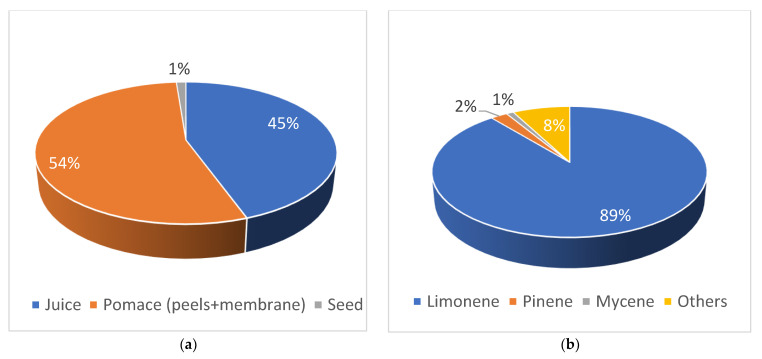
Composition of oranges (**a**) and its bioactive compounds (**b**).

As illustrated in Figure 1, the 2019 global orange harvest exceeded 68 million tons. More than half of the world’s oranges are produced in Asia and the Americas [8]. Approximately 45% of oranges may be processed into orange juice, while the other 54% are classified as orange peels and pomace and are often utilized as animal feed or compost (Figure 2). Since orange pomace and peels are manufactured in enormous quantities and contain a significant proportion of water, they face storage challenges and require immediate attention to avoid putrefaction. The processing of waste into products with additional value minimizes the amount of waste. Therefore, it is a good strategy for the global community. Peels and pomaces have historically been used as animal feed for cows, buffalo, sheep, and goats [9,10]. Orange peel extract also has a strong antioxidant capacity, as well as antibacterial and antiviral properties [11]. It has potential uses in the pharmaceutical and nutraceutical sectors.

Traditionally, Soxhlet extraction and maceration in association with an organic or toxic solvent are used for the extraction of orange pomace [12,13]. The hazardous solvent is incompatible with the extraction of orange pomace and peels, particularly for health and wellness products [14]. Consequently, the quality of the extract is poor. In addition, traditional extraction has a lengthy extraction period and uses high-temperature conditions. Thus, orange peel bioactive compounds are rapidly damaged [15]. Despite this, ‘green extraction’ is usually recognized as a solution to substitute for the conventional extraction method. The purpose of green extraction is to increase yields at a lower price of production. Due to the lack of organic solvents, toxic residue formation is avoided [16,17,18,19]. Thus, important improvements in green technologies such as microwave-assisted extraction (MAE), supercritical carbon dioxide (ScCO_2_) extraction, and subcritical water extraction (SWE) have been developed [20,21,22,23,24,25,26,27,28,29,30,31,32,33,34,35]. Therefore, this mini-review article provides a detailed overview of orange peels, discusses their current extractions, and proposes a direction for future perspectives on orange peel valorization.

## 2. Methods of Review

This short review was conducted from Google Scholar, Scopus, and Web of Science from 2005 to 2022.

## 3. Interest Compounds of Orange Peels and Pomaces

### 3.1. Pectin

Pectin as a colloidal carbohydrate complex is typically located above the intermediate layer of the lamella and main cell walls in a variety of plant species [36]. The majority of commercial pectin comes from apple pomace, orange peels, and banana peels. Orange peels and pomace are accessible in considerable volumes as byproducts of fruit juice [37]. Pectin is mostly derived from tropical and subtropical fruit byproducts, which are predominantly generated by agro-industrial industries especially from orange peels [38]. However, it is important to note that pectin yield and physical properties depend on the separation process utilized and other factors, such as extraction acid, pH, temperature, liquid–solid ratio, and extraction time [7,36,39,40].

### 3.2. Phenolic Compounds

Phenolics are a heterogeneous group of compounds produced by the secondary metabolism of plants and herbs [19]. They have at least one aromatic ring, and one or more hydroxyl groups are covalently attached to aromatic structures. There are two categories of phenolic compounds: non-flavonoid and flavonoid compounds [41]. Flavonoids consist of two aromatic rings connected by an oxygen heterocycle. They may be subclassified as flavanols, flavones, isoflavones, anthocyanins, flavanols, and flavanones. Benzoic and cinnamic acids are two of the most typical phenolic acids. They include stilbenes, tannins, and lignin. In recent years, the food sector has been more interested in phenolic compounds, especially from biomasses using orange peels, due to their health benefits; diabetes, cardiovascular illnesses, and inflammation were less prevalent in those with a diet high in phenolic-rich foods [42]. Thus, the employment of sustainable procedures will be attained by the valorization of biomasses using eco-friendly techniques to produce high-quality extracts.

### 3.3. Limonene

Limonene is a terpene in nature and a bioactive compound of various essential oils. It occurs as two optical isomers including a l-limonene and a racemic mixture [43]. It is commonly utilized as a taste and fragrance ingredient in food products such as fruit juices, sweets, chewing gums, soft drinks, and ice creams. This is due to its pleasant lemon-like odor [44]. It is also an affordable compound used in cosmetics such as soaps, perfumes, shampoos, and shower gels [45]. It is also categorized as safe for food preservation [46]. Therefore, the extraction of limonene from orange pomace as a waste will enhance the economic value of the pomace as a high-end product. Therefore, the utilization of sustainable processes will be achieved with the valorization of biomasses using green processes to obtain high-quality extracts. 

## 4. Various Extraction Method for Orange Pomace/Peels Valorization

**Figure 3:**
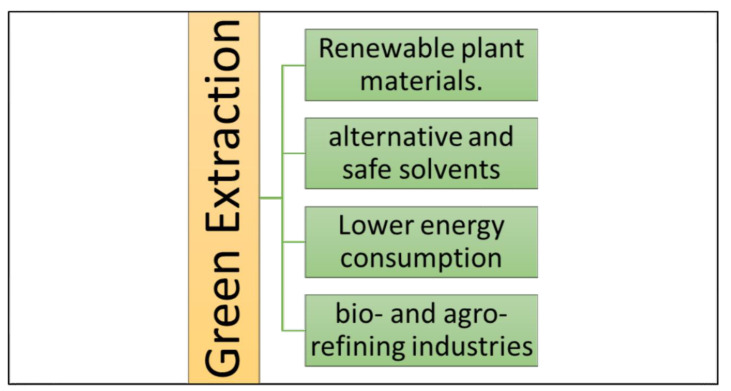
Four principles of eco-friendly extraction.

Extraction is the biological or chemical method of separating components from natural materials. In an attempt to preserve the environment, the international community has recently implemented green extraction. This concept is based on processes that consume less energy, alternative solvents, renewable resources, and safe and high-quality extracts [47]. It is considered the most effective alternative to traditional solvent extraction methods for bioactive compounds. There are four principles of eco-friendly extraction, as shown in Figure 3 [48]. Furthermore, Table 1 shows the benefits and drawbacks of various extraction methods.

In order to valorize orange pomace/peels, green technology provides greener and more sustainable processes to obtain high quantities and qualities of orange peels with the minimum production of waste. There are current extraction methods that can be applied to extract palm oil, including MAE, UAE, SWE, and ScCO_2_ extraction. These procedures have several drawbacks, including a higher operating cost, a lower yield, a greater energy need, and the use of inorganic solvents. Due to the existence of traces in the food matrix, the latter are accountable for poor health impacts, including bad effects on the human body [57]. 

### 4.1. Microwave-Assisted Extraction (MAE)

Green extraction has gained attention for reducing the use of solvents and decreasing energy consumption. Therefore, MAE has been developed as a unique extraction technique for bioactive compounds, especially for limonene. This procedure consumes less time and energy consumption, resulting in a greater extraction yield. In addition, it has been used with traditional techniques (steam distillation, steam diffusion, hydrodistillation, and air hydrodistillation) to simplify the process and improve its efficiency [57]. Therefore, the application of MAE to orange pomace/peels is suitable as a sustainable extraction method. When processing food and food-related matrices, non-ionizing electromagnetic radiations are used, with a frequency range of 300 MHz to 300 GHz [58]. 

Heating in MAE is targeted and selective, with almost no heat wasted on the environment, as it is conducted in a closed system [59]. Microwave heating is significantly influenced by the polarity of the materials/solvents [58]. Based on the electrophoretic transport of ions and electrons, these electromagnetic radiations create an electric field by commencing ionic conduction. This induces dipole rotation, which aligns molecules with the electric field and heats the substance. This heating leads water molecules to evaporate. Pressure is generated inside the plant cell, causing the cell walls to rupture and facilitating the release of bioactive compounds [53,57,60,61]. The optimal condition of orange peels and pomaces are shown in Table 2. 

Prakash Maran, Sivakumar, Thirugnanasambandham and Sridhar [62] employed MAE to extract pectin from dried orange peels. The quantity of extracted pectin rose with increasing microwave power but decreased with increasing duration, pH, and solid–liquid ratio. The absorption of microwave radiation in the extraction system boosted the thermal accumulation of the solvent system, resulting in the dissolving of pectin into the solution until 125 s [66]. However, long-term exposure to microwave radiation may result in the destruction of pectin. Moreover, a less acidic extraction solvent is capable of directly contacting the insoluble pectin and promoting the hydrolysis of the insoluble pectin components into soluble pectin, hence boosting pectin recovery [67]. Pectin yields improved with increasing solid–liquid ratios up to 1:16 g/mL, with the greater amount of extraction solvent producing excessive swelling of the materials and the materials absorbing microwaves directly. Therefore, the cell walls were compromised, resulting in the effortless release of pectin into the surrounding media. However, when the solid–liquid ratio surpassed 1:16 g/mL, the solution became saturated with the solute, which adversely impacted the mass transfer rate, impeded the pectin’s penetration into the solution, and reduced the extraction yield [68].

Kute, Mohapatra, Babu and Sawant [63] carried out the MAE of pectin from dried orange peel powder with acidified water using nitric acid as a catalyst. Reducing the pH values from 2.5 to 1.5, the pectin yield rose from 9.55% to 15.79% with the solvent. The less acidic extraction solvent was also capable of entering direct contact with insoluble pectin and promoting the hydrolysis of insoluble pectin elements into soluble pectin, hence enhancing pectin recovery. The solvent (water) can efficiently absorb microwave radiation and lead to improved swelling of plant material, which is advantageous due to the increased contact surface area between the plant matrix and the solvent. Furthermore, increasing the surface area may promote pectin recovery. By increasing the microwave irradiation energy, the penetration of the solvent into the plant matrix may be boosted and can efficiently deliver solvent to plant cells for pectin extraction. The interaction of molecules with an electromagnetic field enables a quick transfer of energy to the solvent and matrix, hence facilitating the extraction of components by dissolution. As a polar solvent, water can absorb microwave radiation effectively, resulting in effective heating. Moreover, the microwave irradiation accelerates cell rupture by spontaneous increases in temperature and internal pressure within the cells of the plant sample, which promotes the destruction of the sample surface and, in turn, causes the exudation of pectin within the plant cells into the surrounding solvents and increases the extraction yield.

Hosseini, Khodaiyan and Yarmand [64] employed MAE to extract pectin from sour orange peel. The influence of various liquid-to-solid ratios (ranging from 5 to 45 (*v*/*w*)) on the extraction yield was assessed. The extraction yields rose dramatically from 6.7% to 9.2% when the liquid-to-solid ratio increased from 5% to 15% (*v*/*w*). However, extraction yields fell when the liquid–solid ratio increased further. Up to a liquid–solid ratio of 15 (*v*/*w*), the solvent may improve the contact surface area between the plant matrix and the solvent, hence increasing the pectin extraction yield. However, the larger liquid–solid ratio decreased the microwave adsorption of solid material. This is because the solvent absorbed more energy, resulting in a lower pectin mass transfer rate and extraction yield [69].

Toan, Truc, Le, Quyen and Tran [65] developed a method of extraction of Vietnamese orange peel (*Citrus sinensis*) using MAE assisted by hydrodistillation to obtain the essential oil. As the power increased from 300 W to 700 W, the recovery efficiency dropped, and the maximum oil production was attained at 300 W of microwave power, with a yield of 2%. Increased microwave irradiation increases the roughness and velocity of compound movement, hence aiding the diffusion process. Moreover, warmth denatures and dissolves cell membranes through the activity of gas bubbles, facilitating the separation process. The extraction time depends on the substance, solvent, and temperature of the extraction. Extraction efficiency may be improved by extending the extraction time. However, this relationship remains true only up to a specific period of extraction, beyond which extending the time does not improve extraction efficiency. However, lengthier extraction times diminish the quality of essential oils and use more time and energy.

MAE is a green technique that can be used for the recovery of limonene from orange peels according to a method by Attard, et al. [70]. Due to its great selectivity for limonene, considerably reduced extraction periods, and double the limonene yields of traditional heating, microwave irradiation has been shown to be a more effective approach than conventional heating. Notably, this was not seen with conventional heating, which suggests that microwave radiation interacts favorably with the material during extraction, resulting in simultaneous cell rupture and diffusion and a higher yield. This gives crucial insight into the evolution of orange peel extraction methods.

Benassi, Alessandri and Vassalini [38] also found contradictive results that hot water is more effective (greater extraction yields) and enables the production of pectin from orange pomace compared to MAE. Particularly, when citric acid is added, both extraction yield (21%) and % degree of esterification (82.5%) reaches their highest values. Simultaneously, ethanol use is restricted, and the total energy (instruments and embodied energy of chemicals) necessary for pectin production achieves its minimum value. Therefore, acidic extraction is the most environmentally friendly technique to obtain high-methoxyl pectin. 

### 4.2. Ultrasound-Assisted Extraction (UAE)

UAE seems to be of interest in food manufacturing for the enhancement of compounds of interest from plant sources. Utilizing UAE as a pretreatment step in a unit process has the potential to improve the extraction of aromatic compounds and polyphenolics, such as limonene, pectin, oils, and other bioactive compounds. This method reduces solvent usage, causes a higher yield, and shortens the extraction time, which will be of great industrial interest [71,72]. All of the experiments make use of high-powered ultrasound at frequencies ranging from 20 to 25 kHz. The optimal conditions of orange peels and pomaces for valorization using UAE are shown in Table 3. 

Hosseini, Khodaiyan, Kazemi and Najari [73] discovered a minimal correlation between extraction yield and degree of esterification (DE). The optimal extraction conditions (ultrasound power of 150 W, irradiation period of 10 min, and pH of 1.5) led to the maximum extraction yield, whereas DE was quite low under these conditions. In general, even when the extraction conditions of high power and low pH result in a large yield, the DE for these circumstances is low, most likely owing to the de-esterification of pectin [8]. Compared to prior research, the DE in ultrasound-assisted extraction was lower and greater than in aqueous extraction (23% DE) and microwave-assisted extraction (1.5 0.2% DE). The extraction yield was greatest at the lowest pH value. In other words, the highly acidic conditions enhanced pectin leakage from the plant material, which boosted the orange peel extraction yield. In this investigation, ultrasonic irradiation duration was one of the other significant parameters that had an inverse influence on the extraction yield. This discovery may be attributable to the fragmentation of the pectin structure into oligosaccharides by the cavitation effects of ultrasonic waves during high-exposure periods.

Selahvarzi, Ramezan, Sanjabi, Namdar, Akbarmivehie, Mirsaeedghazi and Azarikia [74] also optimized the conditions to extract phenolic compounds from orange peels. To optimize the extraction of phenolics from orange peels, the influence of three variables, including extraction time (10 to 40 min), temperature (40 to 70 °C), and solid-to-solvent ratio (1:20 and 1:40 g/mL), was examined. Orange peels had the maximum antioxidant activity (54.27%) and total phenolic compounds (1.86 mg/g) at 40 min, 70 °C, and 1:40 g/mL. Tests on beverages revealed that adding pomegranate and orange peels increased their amounts from 10% to 15% *v*/*v* and antioxidant activity. Flavonoids (83.44 mg QE/100 mL) and vitamin C (212.37 mg/100 mL) were more abundant in the beverage containing 15% orange peels than pomegranate peels. By increasing the temperature, duration, and solvent-to-solid ratio of ultrasonic extraction, more phenolic chemicals were extracted from orange peel. The extraction duration had the greatest impact, whereas the ratio of solvent to solids had the lowest phenolic recovery. Increasing the temperature of the ultrasound extraction process increases the extraction efficiency of phenolic compounds from the materials by increasing the solubility of bioactive compounds, improving the solvent penetration rate and mass transfer, and decreasing the surface tension and viscosity of the solvent. Appropriate extraction temperatures diminish cell wall resistance, soften plant tissues, and facilitate the breakdown of linkages between cellular components (proteins or polysaccharides) and phenolic compounds. This has the overall effect of increasing the solubility of phenolic compounds, hence boosting the extraction rate. 

Kaur and Kapoor [77] also determined the influence of various factors on UAE for kinnow mandarin peel as a part of the citrus family. The maximum DPPH radical scavenging activity (64.70%), TPC (36.17 mg/g), and ferric-reducing antioxidant power assay (28.17 mM/100 g) were observed at a liquid-to-solid ratio of 30:1, an amplitude of 31%, and a temperature of 41 °C. Savic Gajic, Savic, Gajic and Dosic [75] developed a UAE method of extraction of orange peels using olive oil as a solvent. The optimal conditions are shown in Table 4. At longer extraction durations, the influence of extraction temperature was more significant. The increase in temperature to 45 °C caused a rise in carotenoids, which subsequently declined significantly. Due to the decrease in viscosity and the rise in the diffusion coefficient, olive oil was better able to permeate plant tissue and dissolve carotenoids because of its heating. During the first step of extraction, the targeted compounds were leached from the injured cell wall surface. The prolonged influence of cavitation energy on cell membranes caused the release of carotenoids. After this interval, the diffusion of carotenoids through the cell walls was also carried out at a significantly slower rate. As the ultrasonic waves passed through the medium, they compressed and sheared the solvent molecules, resulting in localized changes in density and the modulus of elasticity [36]. At a limited distance from the source of the ultrasound, the original sinusoidal waves of compression and shear deformed into shock waves. At the edge of the ultrasonic wave, a dramatic drop in pressure generated little bubbles. In addition to facilitating swelling and hydration, the ultrasound led to an increase in cell wall pores. The enhanced extraction effectiveness was also attributed to diffusion through the cell walls and the leaching of cellular contents. Reduced particle size by ultrasonic disintegration increased the number of cells immediately exposed to solvent and ultrasonic cavitation extraction. 

Feng [14] evaluated and optimized the effects of solvent concentration (50 to 100%), treated time (25 to 85 min), treated temperature (25 to 55 °C), and two categorical factors: ultrasonic frequency (28 kHz or 40 kHz) and type of solvent (methanol and ethanol) on yield from waste orange peels. The solvent concentration had a linear impact on the crude extraction and precipitation yield. Utilizing methanol at 55 °C, with a 40 kHz ultrasonic frequency, and for an 85 min extraction time, the best and practicable UAE conditions for improving the yield (61.42%) were achieved. The yield was influenced by the interaction of time and temperature. The effects of solvent concentration were intensified as ultrasonic frequency increased. It was discovered that methanol extract produced the maximum extraction yield from sweet orange peel (15.56 0.60 g/100 g). Methanol was found to be more effective than other organic solvents, such as hexane, petroleum ether, and acetone, for extracting phytochemicals from orange peels. The polarity of the solvents used for extraction may contribute to this occurrence. The methanolic extract had a greater extraction yield, indicating that a highly polar solvent increases extraction efficiency. Methanol and ethanol have relative polarities of 0.76 and 0.65, respectively.

Hundie [76] discovered that a citric acid pH solution was a crucial influence in orange peel valorization utilizing UAE. Likewise, the interaction between pH, solution vs. ultrasonic power, and pH solution vs. liquid–solid ratio had a significant impact on the extraction yield. In addition to the liquid-to-solid ratio and pH solution, the interaction between pH and ultrasonic power had a significant impact on pectin extraction. The maximum extraction yield was achieved at the lowest pH solution and greatest ultrasonic power. This indicates that very acidic conditions caused pectin to flow from the plant material, hence increasing the extraction efficiency. Ultrasound power was another effective variable that had a direct effect on the extraction [78]. This finding may be attributable to the cavitation effects of ultrasonic waves, which improve the solvent penetration into the intercellular substance of the plant. Irradiation duration is one of the crucial process factors that greatly affects the pectin yield compared to the liquid-to-solid ratio. In the first phase, the extraction efficiency was raised to 22.5 min due to the formation of cavitation bubbles by ultrasonic waves, which aid in the rupture of the plant cell wall to boost the extraction efficiency of pectin and create swelling and hydration of the plant material. The yield of pectin was not significantly affected by the liquid–solid ratio compared to other factors [79]. Variation of the experimental range above a solid-to-liquid ratio of 1:2.5 g/mL may lead to a different result about the role of this working parameter in the cavitation effect happening in an extraction aided by ultrasonic technique and, subsequently, its influence on the extraction efficiency.

### 4.3. Supercritical Carbon Dioxide (ScCO_2_) Extraction

ScCO_2_ extraction provides a green recovery extract based on the high purity of solutes. Low-temperature conditions provide higher amounts of bioactive compounds. This is because the solubility of the compounds can be adjusted based on pressure and temperature conditions [80,81]. The higher the temperature and the lower the density of CO_2_, the higher the vapor pressure of compounds [82]. Moreover, the solubility of compounds will increase with increasing pressure [80,83]. This method has a limitation in compound interest in that it is suitable for non-polar compounds. The addition of a polar cosolvent or modifier to CO_2_ can increase the solvent power and selectivity to extract the polar compounds [84]. The modifier’s swelling effect on the raw material will increase the contact surface area between the raw material and the solvent [85]. Ethanol is a preferable modifier due to its availability in the food-grade class. The optimal condition of orange peels and pomaces valorization using ScCO_2_ are shown in Table 4.

**Table 4:** The optimal parameters and outcomes in orange peel extraction by ScCO_2_.

Optimal Conditions	Outcomes	Source
28.7 MPa and 60 °C	Hesperidin, apigenin, quercetin, cyanidin, p-coumaric acid, ferulic acid, and sinapic acid were found to be raised between 2 and 260 times by ScCO_2_ extraction.	[86]
400 bar, 50 °C, and 10% ethanol	The TPC and antioxidant activity were 19.5 1.8 mg/g and 21.93 mol/100 g.	[87]
20% ethanol, 30 MPa, and 60 °C	The highest content of naringin was 35.26 mg/g. The highest antiradical activity was 31.78–59.51 µmolTE/g.	[88]
35 MPa and temperatures of 40 °C using pure ethanol	The maximum amounts of essential oil and TPC were 2.62% and 21.8 mg GAE/g dry extract, respectively.	[89]
313.15 K to 323.15 K and to 300 bar	The maximum TPC was 35 mg/g extract.	[90]

Argun, Argun, Arslan, Nas, Ates, Tongur and Cakmakcı [86] revealed that citrus processing factories generate large amounts of wastewater containing considerable environmental pollutants, many of which are bioactive compounds. The objective of this work was to recover important bioactive components from orange manufacturing wastes using supercritical CO_2_ extraction. The optimal experimental conditions for maximal extract production, total phenolic content, total flavonoid content, and antioxidant activity were determined to be 28.7 MPa and 60 °C. Some important phenolic compounds, such as hesperidin, apigenin, quercetin, cyanidin, p-coumaric acid, ferulic acid, and sinapic acid, were found to be raised between 2 and 260 times by ScCO_2_ extraction. The TFC values of the extracts were found to increase with increasing temperature and decrease with increasing pressure. It was established that the optimal extraction conditions for the TFC values are 20 MPa and 60 °C. High-molecular-weight flavonoids have less polarity than other phenolics; hence, they should be more soluble in low-density CO_2_ under conditions of low pressure and high temperature. 

In orange peel valorization, essential oil was extracted using ScCO_2_ with ethanol as cosolvent [87]. Mandarin peel contains polyphenols that may be extracted to increase the value of this byproduct. Supercritical fluid extraction is an effective and environmentally benign method for the recovery of these bioactive compounds and is also suggested for the extraction of essential oils from orange peel and other citrus fruits. The extraction conditions were pressures of 300 and 400 bar and temperatures of 40 and 50 °C, respectively, for the response of TPC and antioxidant activity. The optimal extraction conditions were achieved at 400 bar, 50 °C, and 10% ethanol. The TPC and antioxidant activity were 19.5 1.8 mg/g and 21.93 mol/100 g, respectively. Seven phenolic compounds were discovered, with hesperidin being the primary flavonoid component. A higher modifier ratio will increase the yield and recovery of phenolic compounds from orange peels. 

The use of ethanol enhanced the TPCs’ extraction. These results matched those of Espinosa-Pardo, Nakajima, Macedo, Macedo and Martínez [89], who observed an increase in phenolic compounds in the extract of dry orange pomace produced by supercritical fluid extraction with CO_2_ and a minor quantity of ethanol as a cosolvent (6%). The TPCs were greater than those of the dry orange pomace extract analyzed by Espinosa-Pardo, Nakajima, Macedo, Macedo and Martínez [89], who found TPCs ranging from 18.0 to 21.8 mg of GAE per gram of extract, depending on the extraction pressure and temperature. This finding may be attributable to the extended extraction period (5 h) used in our trials compared to the 75 min employed by Espinosa-Pardo, Nakajima, Macedo, Macedo and Martínez [89]. In fact, the extraction duration might affect the content of the extract. Moreover, the initial phenolic content of the various matrices analyzed, pomace and peels, might vary according to the results on the effect. However, the findings were comparable to those of Benelli, Riehl, Smânia, Smânia and Ferreira [90], who extracted orange pomace for 300 min to obtain TPC concentrations ranging from 30 to 35 mg GAE/g.

In addition, raising the pressure to 30 MPa and adding 20% ethanol led to an increase in the TPC content of all extract types. High pressure alters the distribution and aggregation of phenolic compounds and increases the penetration of the solvent into cells by disrupting cell walls and hydrophobic bonds in the cell membrane, which can result in increased permeability and the release of antioxidant components [91]. Romano, De Luca, Aiello, Rossi, Pizzolongo and Masi [88] also studied the valorization of citrus/orange peels using ScCO_2_. Evaluations were conducted on the extraction yield and TPC. These results indicate that extraction using a combination of liquid CO_2_ and ethanol may be a viable alternative extraction method, using 80% less organic solvent and yielding extracts with a high concentration of volatile organic compounds. The presence of ethanol enhanced the extraction of TPCs from all matrices, with the exception of tangerine when supercritical CO_2_ at 20 MPa was utilized. An increase in phenolic compounds in the supercritical fluid extraction of dry orange pomace was seen with CO_2_ and a minor quantity (6%) of ethanol as a cosolvent. In fact, the extraction duration might affect the contents of the extract. Additionally, the initial phenolic content of the various matrices analyzed, pomace and peels, might vary according to the growing conditions.

### 4.4. Subcritical Water Extraction (SWE)

In light of the significance of functional foods and health care, there has been a growing focus on the active compounds extracted using environmentally friendly techniques. How to assure biological activity and yield recovery throughout the extraction is a pressing issue that requires immediate resolution. SWE offers an eco-friendly way of extracting bioactive compounds from orange pomace or peels. This method offers higher active compounds and has considerable promise in healthcare and functional foods. In the meantime, health product research should concentrate on customer acceptance, safety, legal considerations, and commercial availability. The transformation of water into subcritical water is achieved by heating water to a pressure sufficient to keep it in a liquid state at a temperature between 100 °C and 374 °C and at a pressure between 1 and 22.1 MPa [40]. An increase in temperature will decrease the viscosity, dielectric constant, and surface tension. Therefore, it will increase the diffusivity of water. At a certain temperature level, an optimal pressure can be applied to keep the water liquid [92,93,94,95]. In addition, the higher pressure may facilitate extraction by allowing water to enter the matrix (pores). The polarity of subcritical water diminishes as the temperature increases. Therefore, polar and non-polar compounds may be extracted from the material [96,97,98,99]. The optimal conditions for orange peel and pomace valorization using ScCO_2_ are shown in Table 5. 

Lachos-Perez, Baseggio, Torres-Mayanga, Ávila, Tompsett, Marostica, Goldbeck, Timko, Rostagno, Martinez and Forster-Carneiro [100] discovered that hesperidin, flavanones, and narirutin compounds present the majority of the naturally occurring compounds in citrus pomaces. The effects of temperature and water flow rate were examined. The impacts of thermodynamics, mass transfer, and thermal degrading on extraction performance were investigated at various flow rates. Specifically, within thermodynamic restrictions (i.e., solubility), extraction yields should rise as the flow rate increases. Similarly, extraction yields of thermally labile components might benefit from an increase in continuous phase flow rates since exposure durations of extracted components to thermally harmful circumstances reduce as flow rates increase. In conclusion, mass transfer restricted recovery performance will be independent of continuous phase flow rate and may potentially decline for inaccessible components. At 10 mL/min and 150 °C, the highest yields of narirutin (21.98 mg/g) and hesperidin (188.74 mg/g) were obtained. These yields included almost 21% of the total flavanones in the extracts, resulting in the cleanest extracts. Comparing SWE to Soxhlet extraction and MAE demonstrates that SWE is a very effective approach to obtaining extracts with stronger antioxidant activity. In addition, it shows that the degradation of hesperidin and narirutin isolated from defatted orange peel by SWE commences at temperatures greater than 160 °C. 

Hwang [101] also discovered that the effectiveness of extracting narirutin and hesperidin from *Citrus unshiu* peel was enhanced by pulsed electric field (PEF). The samples were treated with a 3 kV/cm PEF for 60 and 120 s. SWE was carried out at temperatures ranging from 110 to 190 °C for 3 to 15 min. The maximum concentration of hesperidin was 46.96 mg/g after PEF treatment at 120 s, with a temperature of 150 °C for 15 min, whereas the highest concentration of narirutin was 8.7 mg/g after PEF treatment at 120 s, with a temperature of 190 °C for 5 min. Narirutin and hesperidin rose with the PEF treatment duration. The PEF enhanced the quantity of extracted hesperidin by 22.1% and narirutin by 33.6%, respectively. This research demonstrates the possibility for PEF pretreatment to increase flavonoid recovery from orange peels. The findings demonstrate that combining the SWE and PEF is a successful approach for extracting hesperidin and narirutin from *C. unshiu* peel, particularly considering its primary benefits of permitting quick extraction without the use of organic solvents, which are linked with toxicity and economic problems.

Kim and Lim [102] examined the efficiency of flavonoids extracted from dried orange peels using SWE. The increased yield of citrus flavonoids at higher temperatures may be attributable to the lower dielectric constant and polarity of water, which makes it more favorable for the extraction of less polar compounds [93]. In addition, when the temperature of the water increases, its viscosity drops, but its surface tension and diffusivity increase, resulting in a higher mass transfer rate of the solute from the sample matrix into the solvent [29,30]. The dielectric constant of water decreases from 80.2 at 20 °C to 50.5 and 41.9 at 120 and 160 °C, respectively [104]. The viscosity of water reduces from 1002 106 Pas at 20 °C to 232 × 10^−6^ and 170 × 10^−6^ Pas at 120 and 160 °C, respectively [105]. The surface tension of water reduces from 72.7 mN/m at 20 °C to 56.9 mN/m at 110 °C and 48.3 mN/m at 150 °C. The diffusivity of water rises from 2.3 × 10^−9^ m^2^/s at 25 °C to 9.8 × 10^−9^ m^2^/s at 110 °C and 15.7 × 10^−9^ m^2^/s at 150 °C [105]. Initially, the yield of flavonoids rose at 180 °C, but after 10 min of extraction, it started to decline in comparison to 160 °C. This showed that flavonoids undergo heat hydrolysis or destruction at temperatures over 160 °C. 

## 5. Future Perspectives

Although orange peels and pomace are produced in numerous amounts and contain a large percentage of water, they must be stored in a manner that prevents them from rotting promptly. Processing trash into goods with additional value might decrease waste and become a viable alternative for global society. Pomaces and peels have been used to feed cows, buffaloes, sheep, and goats for a very long time. High quantities of pectin, phenolics, and limonene are extremely advantageous and may be used in the future, especially in the nutraceutical and pharmaceutical sectors. Historically, orange pomaces or peels were extracted using maceration and Soxhlet extraction, both of which were conventional procedures. On the other hand, the green extraction of plant material has surpassed conventional extraction techniques. It has become necessary to increase the output of high-quality extracts that are safe for human health. Therefore, breakthroughs have been achieved in green extraction technologies, including MAE, UAE, ScCO_2_, and SWE. The disadvantage of green extraction is that it cannot be scaled up since continuous methods are preferable. Green extraction methods are also too costly for new and small businesses; thus, careful optimization of the process parameters is necessary. Furthermore, the future perspective is that the valorization of other fruit waste can be carried out using these green extraction methods due to the high quality and quantity of extract. Therefore, sustainable utilization can be achieved using biomass with green processes.

## 6. Conclusions

**Figure 4:**
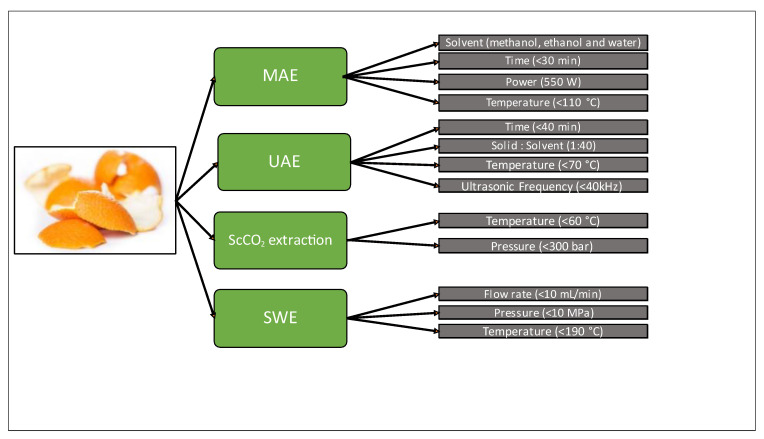
Influenced parameters on the orange peel and pomace valorization using green extractions.

Orange pomace and peels, as agricultural wastes, are a natural source of pectin and limonene. They also include the phenolic compounds that are advantageous to the food industry, including diabetes, cardiovascular diseases, Parkinson’s disease, Alzheimer’s disease, and inflammation. Due to the presence of limonene as a compound of interest, orange peels may be used in part as sources of essential oil. Due to its pleasant lemon-like odor, limonene is commonly used as a flavoring and fragrance component in popular culinary items. Limonene is an affordable fragrance used in cosmetics that may be found in soaps, shower gels, perfumes, and shampoos. As described in this article, MAE, UAE, SWE, and ScCO_2_ extraction are successful innovative solutions for the valorization of orange pomace/peels. The influenced parameters in the valorization of orange peels and pomace are described in Figure 4. ScCO_2_ extraction gives a better-quality extract due to low-temperature conditions during the extraction process. This is due to the bioactive compounds of orange peels and pomace will degrade in high-temperature conditions.

## Figures and Tables

**Table 1:** Comparison of ScCO_2_ extraction with traditional methods.

Parameters	ScCO_2_ Extraction	UAE	MAE	SWE	Ref.
Fabrication cost	High	Low	Low	Medium	[47]
Toxicity	High	Medium	Medium	High	[49,50,51]
Solvent volume	Low (recycled)	Medium	Medium	Medium	[52]
Extract purity	High	Medium	Medium	High	[53]
Selectivity	High	Medium	Medium	High	[54]
Time	Medium	Medium	Fast	Fast	[55]
Compound solubility	High (high pressure)	Medium (no pressure)	Medium (no pressure)	High (high pressure)	[56]

**Table 2:** The optimal parameters and outcomes in orange peel extraction by MAE.

Conditions	Outcomes	Source
169 s, 422 watts, a pH of 1.4, and a solid–liquid ratio of 1:16.9 g/mL	A maximum pectin yield of 19.24%	[62]
pH 1.5, 630 W, 89 s, and a solid–liquid ratio of 1:20.	A maximum pectin yield of 13.32%.	[63]
pH 1.50, 700 W, and an irradiation time of 180 s.	The highest yield of pectin was 29.1%, and DE values of pectin ranged from 1.7% to 37.5%. The LMP of pectin was obtained from these conditions.	[64]
Solid–liquid ratio of 1:3, 300 W, and an irradiation time of 45 min.	The highest yield of essential oil was 2%. Limonene was the major component (98.416%) in orange peels.	[65]

**Table 3:** The optimal parameters and outcomes in orange peel extraction by UAE.

Optimum Conditions	Outcomes	Source
Utilizing methanol for 55 °C, 40 kHz ultrasonic frequency, and 85 min extraction time	The yield was 61.42%.	[14]
150 W, irradiation time 10 min, and pH of 1.5	The maximum extraction yield was 28.07 ± 0.67%. 65.3% of the extracted pectin was galacturonic acid. In addition, the extract’s esterification of 6.77 ± 0.43% was classified as low-methoxyl pectin, which was confirmed by FTIR.	[73]
40 min irradiation time, 70 °C, and solid water ratio 1:40 g/mL.	The maximum antioxidant activity was 54.27%, and total phenolic compounds were 1.86 mg/g.	[74]
35 min irradiation time, 42 ℃, and solid–liquid ratio 1:15 mL/g. Olive oil was used as a solvent.	Carotenoid content was 1.83 mg/100 g dry weight.	[75]
22.5 min irradiation time, pH 1.5, 155 W, and liquid–solid ratio 22.5:1 mL/g	The maximum pectin content was 26.87%.	[76]

**Table 5:** The optimal parameters and outcomes in orange peel extraction by ScCO_2_.

Optimum Conditions	Outcomes	Source
10 mL/min and 150 °C	The highest yields of narirutin and hesperidin were 21.98 mg/g and 188.74 mg/g, respectively.	[100]
150 °C and 15 min	The maximum concentration of hesperidin was 46.96 mg/g after PEF treatment at 120 s with a temperature of 150 °C for 15 min, whereas the highest concentration of narirutin was 8.7 mg/g.	[101]
120–180 °C and 1.0–2.0 mL/min	The extraction yields increased from 40.9, 69.0, and 67.4% at 120 °C to 79.6, 81.9, and 89.0% at 160 °C for hesperidin, narirutin, and PMFs, respectively.	[102,103]

## Data Availability

Data are available upon request from the authors.
